# Causal discovery methods in psychological research: Foundations, algorithms, and a practical tutorial in R

**DOI:** 10.3758/s13428-025-02841-w

**Published:** 2026-02-13

**Authors:** Guangyu Zhu, Li Qian Tay, Mengyan Zhang

**Affiliations:** 1 School of Medicine and Psychology, The Australian National University, Canberra ACT, Australia; 2 Department of Computer Science, University of Oxford, Oxford, UK

**Keywords:** Causal discovery, Causal structure learning, Bayesian network structure learning, Psychological mechanism

## Abstract

Understanding causality and the mechanisms underlying psychological phenomena has been a cornerstone of psychological research with significant implications for theory development and intervention design. While traditional methods such as experimental manipulations or structural equation modelling have been extensively used to explore causal relationships, recent advances in computational techniques have introduced causal discovery methods as a powerful alternative. These methods can uncover complex causal network structures from observational or interventional data, enabling the identification of causal directions in intricate interdependencies involving numerous variables. Building on a growing body of literature, this paper provides a comprehensive survey of core causal discovery algorithms and their recent applications across various disciplines, with a particular focus on their use in uncovering psychological mechanisms. To complement this overview, we provide a tutorial using data from the Health Behavior in School-Aged Children (HBSC) study. This case study demonstrates how causal discovery can be applied to examine gender-specific mechanisms underlying bullying-related outcomes. We also discuss the opportunities and challenges of integrating causal discovery into psychological research.

## Introduction

Understanding causality between variables and mapping out the mechanisms underlying psychological phenomena have long been goals of psychological research. Researchers aim to identify the causal networks that drive specific behaviors or trigger social events (Fung et al., 2023). They also seek to understand the dynamics within such networks, such as how changes in one variable might influence others and the cascading effects these changes could produce (Vallacher et al., 2015). These insights are crucial for theory development and the design of interventions (Bronstein et al., 2024; Vowels, 2022).

To this end, researchers have traditionally relied on laboratory experiments, regression models, and structural equation modeling (SEM), but advances in causal inference over the last years have led to the development of an alternative approach—causal discovery methods. Causal discovery methods seek to uncover causal structures from observational or interventional data (Zanga et al., 2022). These methods have been widely applied across various fields, including genetics (Glymour et al., 2019; Neto et al., 2010), physics (Li et al., 2020), economics (Chong et al., 2010), neuroscience (Biswas & Shlizerman, 2022), pathophysiology (Shen et al., 2020), and epidemiology (Petersen et al., 2024). Recently, they have also been introduced into psychological research, addressing topics related to academic performance (Quintana, 2023) and mental illnesses (Anderson et al., 2023; Bird et al., 2019; Briganti et al., 2021; Černis et al., 2021; Kuipers et al., 2019; Park et al., 2024; Pierce et al., 2023; McNally et al., 2017a, 2017b).

Causal discovery methods serve a unique purpose compared to other approaches, highlighting their distinct value in the broader field of psychology. First, causal discovery can efficiently filter potential causal relationships from large-scale observational data. Randomized controlled experiment has been the “gold standard” for studying causal relationships. However, experiments typically focus on relationships between only a small selection of variables. In contrast, causal discovery methods can simultaneously reveal causal structures in complex networks involving dozens of variables. While methods such as SEM can uncover causal structures between variables, they are more reliant on correlations between variables. In contrast, causal discovery methods can leverage specialized causality criteria to determine the directionality of causal effects from data. Indeed, when Shen et al. (2020) compared the ability of two causal discovery methods (fast causal inference and fast greedy equivalence search) with SEM in recovering a predefined causal graph from data, they found that the causal discovery methods achieved higher true-positive rates, fewer false positives, and were capable of recovering the directionality of edges.

Causal discovery methods can help identify the core causal structures underlying variables of interest and facilitate the exploration of potential subgroups or individual differences in these structures. For example, Fung et al. (2023) applied these methods to investigate factors influencing COVID-19 vaccination intentions and identified several indirect pathways linking vaccination intentions and political affiliation, mediated by factors such as the perceived danger of COVID-19 and the perceived likelihood of contracting the virus. McNally et al. (2017a) identified interference and distress associated with obsessions as a comorbidity factor for both obsessive–compulsive disorder and depression. Moreover, Anderson and colleagues (2023) used these methods to explore personalized causal networks related to three eating-disorder patients, revealing a unique causal pathway leading to each individuals’ symptoms. Such findings open up new possibilities for refining theoretical frameworks that account for subgroup and individual differences, as well as for developing precision interventions that target them.

Causal discovery methods also have the potential to aid the development of a cross-domain framework of human psychology. Understanding the associations between different cognitive processes, as well as their connections to genetic, environmental, and neurophysiological factors, has long been a challenge across disciplines (König et al., 2013). Causal discovery methods, due to their flexibility in accommodating diverse data types, offer a powerful approach to this end. For example, Sokolova and their colleagues (2015) used causal discovery methods to compare the causal network between normal people and ADHD patients, revealing a gene–brain–cognition causal network among DAT1 gene, striatal activation, ADHD symptoms, and smoking behavior. Likewise, Sahin-Ilikoglu et al. (2024) used these methods uncovering a causal network among psychosis proneness, cognitive function (e.g., working memory), brain function (e.g., dysfunction in dorsolateral prefrontal cortex), and social factors (e.g., social cohesion). These examples highlight the potential of causal discovery to unify psychological, biological, and social domains under a coherent causal framework.

To date, several introductions to causal discovery methods have emerged across disciplines such as computer science (Guo et al., 2020; Malinsky & Danks, 2018; Moraffah et al., 2021; Spirtes & Zhang, 2016; Schölkopf et al., 2021; Vowels et al., 2022), economics (Huber, 2024), genetics (Glymour et al., 2019), medical science (Zanga et al., 2022), and psychopathology (Briganti et al., 2022; Briganti et al., 2023), each offering valuable insights into the theory and application of these approaches. Although these methods have attracted growing interest in psychology—particularly in the fields of psychopathology and clinical research (Briganti et al., 2023; McNally et al., 2017a, 2017b)—their application in broader fields such as developmental, educational, and cognitive psychology remains limited.

The reasons may be twofold. First, the current application of causal discovery methods has been largely restricted to identifying group-level mechanisms. Much less attention has been given to their potential for detecting differences in causal structures across subgroups—for example, by age, gender, or cognitive profile. As a result, the contribution of these methods to understanding heterogeneity in psychological processes remains underdeveloped. Second, many tutorials emphasize the technical procedures of causal discovery but pay relatively little attention to how the results can be validated, interpreted, and integrated into existing theoretical frameworks. This lack of attention to validation may contribute to doubts about the reliability and usefulness of these methods in psychological research.

In light of these gaps, the present paper aims to bridge recent developments in causal discovery with current directions in psychological research. The structure of this paper is as follows. The first section will introduce the fundamental concepts underlying causal discovery methods. The second section will focus on the details and rationale behind each causal discovery algorithm. The third section moves beyond existing tutorials by presenting a hands-on tutorial in R. It demonstrates how to apply causal discovery algorithms to psychological data, explore subgroup differences in underlying mechanisms, and validate and compare results across different algorithms. Finally, the concluding section will discuss the opportunities and challenges of applying causal discovery methods in psychology.

## What is causal discovery?

Causal discovery methods aim to uncover the causal structure from a set of observational or interventional data (Zanga et al., 2022). These methods are also referred to as causal structure learning (Kalainathan et al., 2020; Heinze-Deml et al., 2018; Squires & Uhler, 2023) or Bayesian network structure learning (Scanagatta et al., 2019; Scutari & Ness, 2012). Causal discovery methods are data-driven but can also incorporate theoretical or timing prior to forbid or enforce causal relationships between certain variables (Hasan & Gani, 2022).

**Fig. 1:**
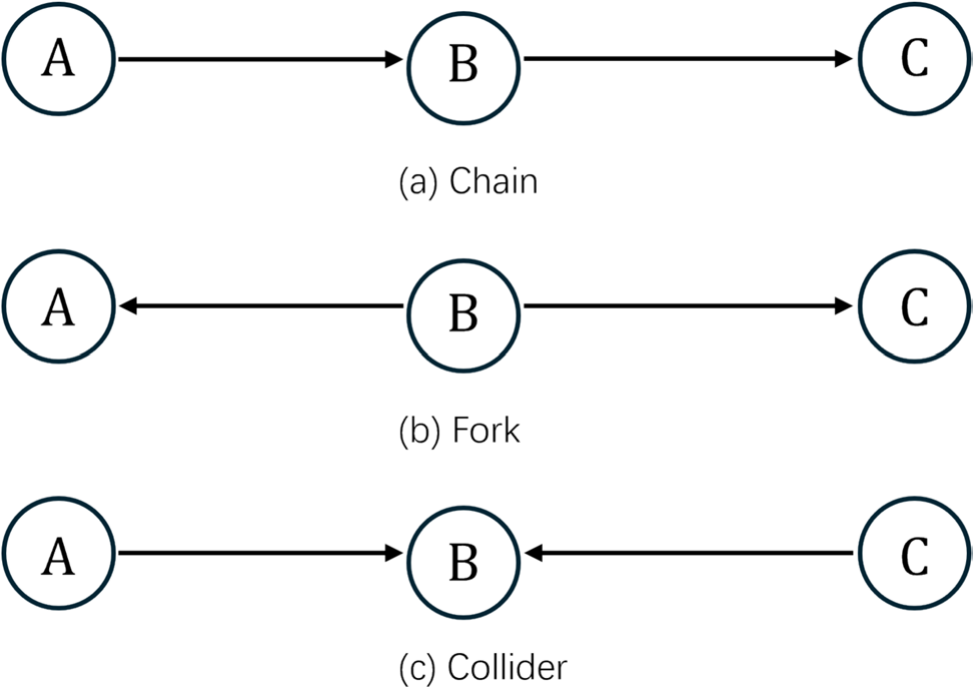
Type of relationships between three variables. *Note.* Nodes represent variables, and directed edges represent causal effects

Causal discovery methods are based on causal graphs, which are graphical representations that summarize researchers’ knowledge and beliefs about the data-generating process (Feng, 2024). 1 presents a glossary of relevant terms. In a causal graph (see Fig. 1), there are two fundamental components: *nodes* (variables) and *edges* (lines connecting the variables). The direction of the edges is indicated by arrows, which represent the direction of a causal effect. In such a graph, the causal effect suggests that the change in a cause will be transmitted to its effect, but the change in an effect cannot be transmitted to its cause (Wright, 1921). Different forms of causal graphs, such as directed acyclic graphs (DAGs), completed partially directed acyclic graphs (CPDAGs), and partial ancestral graphs (PAGs), share the basic representations of causal effects, but vary in their representation of the existence of potential confounders and the direction of causal effect (see Table 2).

**Table 1:** Glossary

	Term	Explanation
Terms for describing causality	Causal relationship	A relationship between two variables where the change in one (the cause) induces a change in the other (the effect)
	Causal pathway	A specific sequence of causal relationships through which one variable affects another, often including intermediating variables
	Causal network	A graphical model showing causal relationships among multiple variables
	Causal structure	An organized pattern of cause-and-effect relationships among variables within a system
Basic concepts for causal discovery	Adjacent pairs	Pairs of nodes directly connected by an edge, showing a direct relationship
	Conditional independence	Variables A and B are conditionally independent on a third variable C, if ${\mathrm{P}}\left({\mathrm{C}}\right)\mathrm{=}{\mathrm{P}}\left({\mathrm{C}}\right){\mathrm{P}}\mathrm{(}{\mathrm{B}}\mathrm{|}{\mathrm{C}}\mathrm{)}$
	D-separation	A concept used to determine whether two sets of variables are independent, given a set of observed variables.
	Equivalence classes	A set of equivalent solutions to the problem of discovering causal relationships from observational data
	High-dimensional data	Data with a large number of variables relative to the number of observations
	V-structures	A configuration in a DAG where two nodes point to a third node, indicating the conditional dependence of them given the third node
	Skeleton	The undirected version of a causal graph
Assumptions behind causal discovery algorithms	Markov property	Every node in a network is conditionally independent of other non-adjacent nodes given the parent nodes
	Acyclicity	Causal relationship that starts at a node should not follow the directed edges back to the same node
	Faithfulness	Observed conditional independences accurately reflect the causal structure
	Sufficiency	All relevant variables are included in the model, with no unobserved confounders or selection bias
Terms related to the evaluation of causal discovery methods	True positive	The number of causal relationships that exist in the true model and are detected by the algorithm
	False positive	The number of causal relationships that do not exist in the true model but are detected by the algorithm
	True negative	The number of causal relationships that do not exist in the true model and are not detected by the algorithm
	False negative	The number of causal relationships that exist in the true model but are not detected by the algorithm
	Precision	A criterion to evaluate the accuracy of causal discovery algorithms to identify causal relationships. Precision = True Positive/(True Positive + False Positive)
	Specificity	A criterion to evaluate the ability of causal discovery algorithms to exclude non-causal relationships. Specificity = True Negative/(True Negative + False Positive)
	Sensitivity	A criterion to evaluate the ability of causal discovery algorithms to identify all causal relationships in the true model. Sensitivity = True Positive/(True Positive + False Negative)

**Table 2:** Different types of causal graphs

Type	Allow for potential confounders	Presentation of causal effect
DAG	No	(1) Directed edges (X → Y) indicate X cause Y
CPDAG	No	(1) Directed edges (X → Y) indicate X cause Y (2) Non-directed edges (X o-o Y) indicate either X cause Y or Y cause X, but the exact direction cannot be determined from the current data
PAG	Yes	(1) Directed edges (X → Y) indicate X cause Y (2) Non-directed edges (X1 o-o X2) indicate either X causes Y, Y causes X, or there is an unobserved confounder influencing both, but the exact direction cannot be determined from the current data (3) Bidirected edge (X1 ↔ X2) indicate neither X cause Y nor Y cause X, but rather that an unobserved confounder causes both (4) Edge with one side arrow and one side cycle (X1 o→ X2) indicate either X causes Y, or there is an unobserved confounder that causes X and Y

In a causal graph, the relationships between each two variables (or nodes) are defined as: (1) two nodes are *adjacent* pairs if they are connected by an edge; (2) two nodes are *non-adjacent* pairs if they are not directly connected by an edge; (3) A is the *parent* of B if A has a causal effect on B; (4) A is the *child* of B if B has a causal effect on A; and (5) if A has a causal effect on B, which in turn influences C, A is considered an *ancestor* of C.

The relationships between three nodes (variables) can be categorized into three types: (1) a *chain* structure, where the causal effect of one variable on another is mediated by the third (e.g., A→B→C); (2) a *fork* structure, where one variable causes the other two (e.g., A←B→C); and (3) a *collider* structure, where one variable is the effect of the other two (e.g., A→B←C).

In the chain and fork relationships (see Fig. 1), conditioning on B blocks the association between A and C, rendering them independent. This phenomenon is known as *conditional independence* or *d-separation* (Geiger et al., 1990; Hayduk et al., 2003). It is fundamental in many causal discovery algorithms (Spirtes et al., 1995, 2001; Tsamardinos et al., 2006a, 2006b). In contrast, in the collider relationship, A and C are independent without conditioning on B, but they become associated when B is conditioned upon. This difference between collider and other types of relationships is used to determine the direction of causal effects, referring to the *V-heuristic*.

Nevertheless, different causal structures can sometimes produce the same data output, making it difficult for algorithms to determine the true causal structure, such as the case of conditional dependence tests on chain and fork structures. Insufficient knowledge to distinguish the true causal structure may result in *equivalence classes*, where two or more structures are equally plausible under the current data and causal criterion.

Causal discovery methods rely on several key assumptions to identify causal relationships between variables (Zanga et al., 2022). First, the causal effects in these causal graphs are typically assumed to follow the Markov property, meaning that a node is conditionally independent of all non-adjacent nodes given its parents. This assumption allows the joint probability distribution of a variable A, P(A), can be decomposed recursively as a product of the probability distribution given its parent variables, ${\mathrm{pa}}\mathrm{(}{\mathrm{A}}\mathrm{)}$:

$$\mathrm{P}\left(\mathrm{A}\right)=\prod \mathrm{P}\left(\mathrm{A}\left|\mathrm{pa}\left(\mathrm{A}\right)\right.\right)$$

Second, the *sufficiency* assumption requires that all relevant variables are included in the analysis and there are no unobserved confounders. A violation of this assumption can induce spurious correlations between observed variables. The requirement about the absence of unmeasured confounding behind the sufficiency assumption may be difficult to satisfy in some psychological studies as it is often difficult to account for all potential confounders (Christenfeld et al., 2004; Rohrer, 2018; Park et al., 2024). However, this assumption is also required when using regression or SEM to estimate the causal relationship between variables. Meanwhile, some causal discovery algorithms can work effectively under the violation of such an assumption (Chen et al., 2021; Glymour et al., 2019; Tashiro et al., 2014).

Third, the *faithfulness* assumption requires observed data attributes, such as conditional independencies, to accurately reflect the causal structure. Violations of such assumption can occur when the causal effects of two paternal nodes cancel out, making a child node appear independent of its parents despite a causal dependency. However, some scholars have also developed robust methods to address situations where this assumption is violated (Isozaki, 2014; Zhalama et al., 2017).

Finally, the *acyclicity* assumption requires that the causal relationship starting at a node should not follow the directed edges back to the same node. That is, there should not be any feedback loops or bidirectional effects. Conceptually, this assumption formalizes the notion that causality has a temporal pattern that moves from causes to effects, and ensures the causal graph is uniquely identifiable from the data. However, some algorithms can overcome this limitation and enable the exploration of cyclic relationships (Richardson, 2013; Park et al., 2024; Strobl, 2019; Spirtes et al., 2013). Additionally, this assumption holds only for cross-sectional data. In longitudinal data, many causal discovery methods can account for causal cyclicity within networks, enabling the representation of causal feedback loops (Chickering, 1996, 2002; Spirtes et al., 2001; Tsamardinos et al., 2006a, 2006b).

## Causal discovery algorithms

Causal discovery algorithms can be categorized into three main types: constraint-based algorithms, score-based algorithms, and asymmetry-based algorithms. Constraint-based algorithms uncover causal structures from data by leveraging conditional independence tests to eliminate impossible causal pathways and applying heuristics, such as V-structures, to decide the direction of causal relationships. Score-based algorithms construct causal networks by maximizing a measure of goodness-of-fit defined as scoring criterion. Asymmetry-based algorithms determine causal relationships between variables by identifying asymmetries between causes and effects, such as independence between variables and residuals. Meanwhile, some hybrid algorithms combine the strengths of these approaches, using constraint-based methods to narrow down possible causal structures and using score-based or asymmetry-based methods to refine the final network. The combination of different types of algorithms can improve its efficiency and accuracy, especially in high-dimensional settings (Tsamardinos et al., 2006a, 2006b). Moreover, researchers have developed methods that integrate causal discovery results from both observational and interventional data (Hauser & Bühlmann, 2012; Ghassami et al., 2019; Jaber et al., 2020; Kalainathan et al., 2020; Kocaoglu et al., 2019; Mooij & Claassen, 2020).

Although a comprehensive review of all algorithms would be beyond the scope for the current tutorial, the following sections introduce several classical causal discovery algorithms to establish the fundamentals of these methods. Readers interested in further details may refer to Zanga et al. (2022) or Vowels et al (2022). The packages and software tools available for implementing these and other causal discovery methods are also summarized in Table 3.

**Table 3:** Causal discovery packages or software

Name	Features	Resource
bnlearn	An R package for Bayesian network structure learning from observational data, including modules for network analysis	https://www.bnlearn.com/
bnstruct	An R package for Bayesian network structure learning from observational data with missing values	https://cran.r-project.org/web/packages/bnstruct/index.html
bnclassify	An R package for learning discrete Bayesian network classifiers from data	https://cran.r-project.org/web/packages/bnclassify/vignettes/overview.pdf
BiDAG	An R package for MCMC-based Bayesian network structure learning, with support for modeling dynamic Bayesian networks	https://cran.r-project.org/web/packages/BiDAG/index.html
dbnr	An R package for modeling and learning dynamic Bayesian networks with support for arbitrary Markov orders	https://cran.r-project.org/web/packages/dbnR/dbnR.pdf
pcalg	An R package for causal discovery based on both observational and interventional data	https://cran.r-project.org/web/packages/pcalg/index.html
Rcausal	An R package containing a range of causal discovery algorithms from the Tetrad software	https://github.com/bd2kccd/r-causal
blip/rblip	An R/Python library for implementing score-based causal discovery algorithms, using BIC and BDeu score functions	https://github.com/mauro-idsia/blip https://github.com/mauro-idsia/r.blip
Causal-Learn	A Python library containing a range of causal discovery algorithms from the Tetrad software	https://causal-learn.readthedocs.io/en/latest/index.html
CausalNex	A Python library integrate machine learning with domain expertise in causal discovery algorithms, enabling robust causal discovery and reasoning	https://causalnex.readthedocs.io/en/latest/
CausalDiscoveryToolbox	A Python library that integrates various causal discovery algorithms from R packages, including pcalg and bnlearn	https://github.com/FenTechSolutions/CausalDiscoveryToolbox
gCastle	A Python library for implementing and evaluating causal structure learning algorithms, providing tools for algorithm performance assessment and comparison	https://github.com/huawei-noah/trustworthyAI/tree/master/gcastle
GOBNILP	A Python/C library for implementing score-based causal discovery algorithms using integer programming	https://benchpressdocs.readthedocs.io/en/latest/
scikit-learn	A Python library for machine learning (ML), including some ML-related causal discovery and causal inference algorithms	https://scikit-learn.org/stable/
trilearn	A Python library for Bayesian structure learning, supporting both discrete log-linear and Gaussian data models	https://github.com/felixleopoldo/trilearn
BANJO	A software application for learning the structure of static and dynamic Bayesian networks	https://users.cs.duke.edu/%7eamink/software/banjo/
Tetrad	A user-friendly software application for causal discovery and inference, requiring minimal knowledge of programming	https://www.ccd.pitt.edu/tools/

Table sorted primarily by programming language and secondarily by the first letter.

## Constraint-based algorithms

*Peter-Clark *(**PC**)* algorithm.* The PC algorithm (Spirtes et al., 2001) uncovers causal structures from observational data through four steps. It first builds a graph with a complete set of variables, each connected by undirected edges. Secondly, the algorithm assesses conditional independence between pairs of variables, conditioned on a set of other variables. If two variables are conditionally independent given a third set of variables, the edge between them is removed, but the connections involving the conditioning variables are retained. This process continues until no more untested adjacent pairs remain, producing the skeleton of a causal network. In the third step, the algorithm applies V-structures (A → B ← C) to triples of variables where A and B, and B and C, are adjacent, but A and C are not, if B is not part of the conditioning set that renders A and C independent. Finally, the remaining edges are oriented to ensure no additional V-structures are formed, resulting in a CPDAG.

The PC algorithm relies on strong assumptions regarding acyclicity, faithfulness, and sufficiency (Zanga et al., 2022). The algorithm is also sensitive to noise and order of variables in the dataset; a single incorrect conditional independence test can affect the entire causal structure. However, its variant, the PC stable algorithm, calculated all the conditional independence tests for the current conditioning set size before modifying the graph structure, so that it is not influenced by the order of variables (Colombo & Maathuis, 2014).

The PC algorithm and its variants have proven effective for high-dimensional data (Gao & Cui, 2015; Kalisch & Bühlmann, 2007; Le et al., 2013; Scutari et al., 2019). They can also accommodate various data types, including continuous and discrete variables (Harris & Drton, 2013; Raghu et al., 2018). The statistical power of constraint-based methods depends on several factors, including sample size, the number or types of variables, the alpha level used in conditional independence tests, and edge strengths. With smaller sample sizes, conditional independence tests are more likely to produce both false positives (i.e., detecting non-existent edges) and false negatives (i.e., failing to detect true edges). Lowering the alpha level can improve precision by reducing false positives, but it may also increase the risk of missing true causal relationships. Kalisch and Bühlmann (2007) demonstrated that, as sample size increases, the true-positive rate tends to rise while the false-positive rate declines. Interestingly, Kummerfeld et al. (2024) observed a paradoxical drop in the precision of edge orientation decisions by the PC algorithm when the sample size increased to 400. They suggested that this may be due to an increase in the number of spurious edges detected at this sample size and recommended lowering the alpha level as the sample size increases to control for false positives. Moreover, the presence of discrete variables inflates the parameter space for conditional independence tests, leading to an exponential increase in the required sample size as the size of the conditioning sets grows (Tsamardinos et al., 2003). As the number of conditional independence tests increases, the statistical power of the PC algorithm also diminishes (Li & Fan, 2020).

**Fast Causal Inference (FCI) algorithm.** The causal sufficiency assumption limits the applicability of the PC algorithm in scenarios involving unobserved confounders or selection bias. To address this issue, the FCI algorithm (Spirtes et al., 1995, 2001) takes into account potential confounders. Accounting for potential confounders complicates the rules of deciding the directions of edges and modifies the third and fourth steps of the PC algorithm (see Zhang, 2008, pg.1879 for all the rules). For example, when there are potential confounders between two nodes, the direction of causal effects between them will be marked as undirected. For any two related variables, there is always the possibility of confounders. However, the FCI algorithm can narrow down the number of confounders by identifying the shared structure among variables. This results in a PAG with a limited number of identified confounders, offering a more nuanced representation of the types of causal effects.

One major advantage of the FCI algorithm over the PC algorithm is its ability to identify potential confounders between variables. This makes the FCI algorithm more applicable to psychology research, where it is often challenging to control for all potential confounders (Christenfeld et al., 2004; Rohrer, 2018; Park et al., 2024). Additionally, FCI can be generalized to scenarios involving cyclic causal relationships (Mooij & Claassen, 2020), which gives it a broader application over PC algorithm and score-based algorithms. However, this flexibility may lead to weaker causal conclusions, as some edges may be marked as bidirectional or undirected. Joint causal inference (JCI), a recently developed algorithm, enhances FCI methods by integrating data from various interventional designs (e.g., perfect, imperfect, stochastic, etc.) to refine causal networks (Mooij et al., 2020). Despite its advantages, the FCI algorithm still relies on assumptions about the Markov chain condition and faithfulness (Spirtes et al., 1995). Yet, few studies have systematically investigated the statistical power of the FCI algorithm. However, given that both the FCI and PC algorithms rely on conditional independence tests based on d-separation, their statistical power tends to follow similar patterns (Li & Fan, 2020).

## Score-based algorithms

**Greedy Equivalent Search (GES) algorithm.** The GES algorithm (Chickering, 1996, 2002) aims to find the optimal causal structure that maximizes the goodness of fit to the data, based on criteria such as the Bayesian information criterion (BIC; Schwarz, 1978). It employs a three-phase strategy: forward phase, backward phase, and turning phase. In the forward phase, the algorithm begins with an empty graph and iteratively adds edges that result in the greatest improvement in the score, continuing until no further improvements can be made. In the backward phase, it then progressively removes edges whose removal leads to the greatest score improvement, stopping when no further significant improvement can be achieved. In the turning phase, the algorithm tries to reverse the direction of the arrows, to achieve a higher score (Hauser & Bühlmann, 2012).

The GES algorithm and its variants enable an efficient search for optimal causal structure without the need to exhaustively explore every possible graph, saving both time and computational power. As the GES algorithm tends to be slow when applied to datasets with more than 50 variables, the Fast Greedy Equivalent Search (FGES) algorithm modifies the search strategy, making it even more effective for high-dimensional data (Smith, 2011; Ramsey et al., 2017). However, sometimes GES will return a non-directional edge due to the presence of equivalence classes. Interventional Greedy Equivalent Search (GIES) algorithm (Hauser & Bühlmann, 2012) improves on the efficiency to decide skeleton and directions of edges, by incorporating the causal information provided by the interventional data.

The GES algorithm relies on the assumptions of sufficiency, faithfulness, and acyclicity (Zanga et al., 2022). The original GES algorithm assumes a Gaussian model for continuous data. When data are non-Gaussian or exhibit nonlinear relationships, alternative strategies may improve the performance of the GES algorithms—such as applying a non-paranormal transformation (Ramsey, 2015), discretizing continuous variables to use a discrete BIC (Deckert & Kummerfeld, 2019), or employing kernel-based generalized scores designed for arbitrary distributions (Huang et al., 2018). In addition, researchers may adopt alternative scoring criteria, such as the Bayesian Dirichlet equivalent uniform score (Laborda et al., 2023) or the degenerate Gaussian score (Andrews et al., 2019), to better accommodate diverse data types.”

The statistical power of the GES algorithm and its variants is shaped by scoring functions, sample size, the number of variables, and edge strength (Kummerfeld et al., 2024). Most algorithms rely on the BIC score, which includes a penalty term for model complexity. The relative influence of this penalty term tends to decrease as sample size increases (Kitson et al., 2023). As a result, the algorithm may overfit by adding more false edges, resulting in reduced precision at larger sample sizes. Kummerfeld et al. (2024) observed a corresponding pattern: the precision of the FGES algorithm in recovering edges peaked at around 400 samples and declined with further increases in sample size. They attributed this trend to the diminishing regularization effect of the BIC penalty term as the sample size grows, and argued that sample size planning in score-based causal discovery should be guided by the anticipated minimal detectable edge strength.

## Asymmetry-based algorithms

**Fig. 2:**
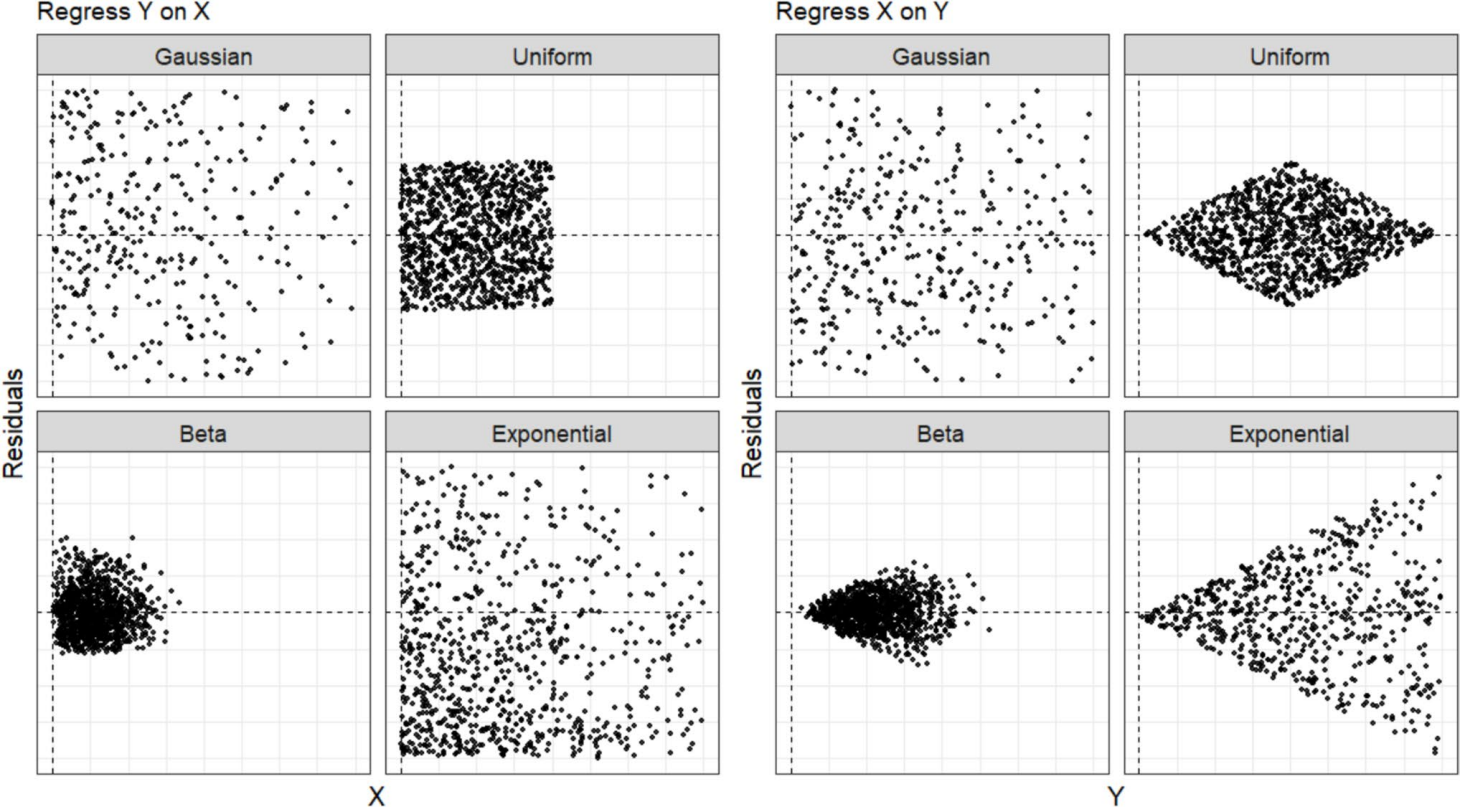
Illustration of causal asymmetry between variables among different types of distributions. The causal relation is X→Y. The left panel displays scatter plots of residuals from a regression model where X predicts Y, plotted against X, while the right panel shows scatter plots of residuals from a regression model where Y predicts X, plotted against Y. In the right panel, the data exhibit a systematic pattern, except for Gaussian distribution

**Linear Non-Gaussian Acyclic Model (LiNGAM).** The LiNGAM (Shimizu et al., 2006) leverages the asymmetry in noise between cause and effect to decide the directions of a causal relationship. Given a cause *X*, effect *Y*, and noise ε, their relationship can be expressed as *Y = bX* + ε, under the assumption of a linear additive model. The independence between *X* and ε holds in only one direction (X → Y) in this linear additive model, which allows the joint distribution between *X* and ε to be used to infer the causal direction (see Fig. 2).

When both *X* and ε are normally distributed, regressing *X* on *Y* and regressing *Y* on *X* produces the similar residual patterns, making it difficult to decide the direction of a causal relationship; but when either *X*, ε, or both deviate from a normal distribution, the asymmetry of noise could be identified from the patterns of residual, enabling the algorithm to decide the direction of a causal relationship (Glymour et al., 2019). This identification process is achieved via independent component analysis, a computational method for separating a multivariate signal into additive subcomponents (Hyvärinen et al., 2001).

The LiNGAM is a linear, non-Gaussian model, which represents both its major strength and limitation. It is designed for continuous variables that deviate from a normal distribution, but this also restricts its application to cases involving normally distributed or discrete variables. This seems to be a drawback for psychologists, as much of psychological data is assumed to be normally distributed, allowing for the use of methods like OLS regression or ANOVA. However, Cramér’s decomposition theorem proposes that a Gaussian distribution can only be calculated as the sum of the other two Gaussian distributions, indicating that true Gaussian distributions are less common in real-world settings (Cramér, 1970). Supporting this, an analysis of 693 distributions from psychological studies found that only 5.5% were strictly normal (Blanca et al., 2013). Therefore, LiNGAM remains highly applicable in psychology.

LiNGAM assumes linearity, faithfulness, and acyclicity. While the linearity assumption can be relaxed by modeling non-linear relationships while maintaining independence between x and e (Hoyer et al., 2008), the causal faithfulness assumption can be extended using ICA LiNGAM (Hoyer et al., 2008) or DirectLiNGAM (Shimizu et al., 2011).

For the original LiNGAM and its variants, power to recover edges and to estimate their strengths, depends on sample size, the number of variables, and edge strength (Leyder et al., 2025; Prakash et al., 2024; Tashiro et al., 2014). For example, Tashiro et al. (2014) suggested that for medium-sized graphs (5 ≤ number of edges ≤ 30) with moderate edge strengths, achieving a precision and recall above 0.85 typically requires a sample size of at least 1000 for the classic LiNGAM, and around 500 for latent-robust variants such as ParceLiNGAM. Moreover, statistical power tends to decrease as the number of variables or edges increases and as edge strength weakens. These effects are further amplified in the presence of heavy-tailed noise and latent confounding (Leyder et al., 2025).

## Hybrid algorithms

**Max-Min Hill–Climbing (MMHC) algorithm.** The MMHC algorithm (Tsamardinos et al., 2006a, 2006b) combines the strength of constraint-based and score-based algorithms. It starts with the Max-Min Parents and Children algorithm, which involves conditional independence tests to decide the skeleton of causal structure (Tsamardinos et al., 2003). Then, once the skeleton is decided, it applies a score-based search called Hill-Climbing to map out the direction of edges (Selman & Gomes, 2006).

MMHC algorithm also requires assumptions of sufficiency, faithfulness, and acyclicity (Tsamardinos et al., 2006a, 2006b). Regarding performance, the MMHC algorithm has been shown to outperform the PC algorithm, GES algorithm, and many others in terms of execution time, number of statistical tests, goodness of fit, and structural hamming distance, across sample sizes ranging from 500 to 5000 (Tsamardinos et al., 2006a, 2006b). It also demonstrates strength in handling high-dimensional datasets. The ability of MMHC algorithm to recover edges also varies with sample size, the number of variables, and edge strength (Park & Park, 2018; Xu et al., 2019). For example, Xu et al. (2019) found that the statistical power of the MMHC algorithm increases rapidly with the first few hundred samples, but then exhibits diminishing returns, stabilizing when the sample size reaches approximately 5–10 times the number of edges in the true graph. Based on these findings, it is generally recommended to maintain a linear increase in sample size with respect to the number of variables to preserve adequate statistical power.

## Tutorial

In this section, we will use a real-world dataset “Health Behavior in School-Aged Children (HBSC), 2005–2006” (Iannotti, 2012) to demonstrate how to run causal discovery algorithms in R. This dataset can be downloaded from: https://www.icpsr.umich.edu/web/NAHDAP/studies/28241. This dataset includes responses from 9227 students across 50 countries worldwide. The survey content covers various topics related to the health of school-aged students, including their daily behaviors, family conditions, friendships, experiences of bullying victimization, and school performance. The research questions for this tutorial are: “What are the causes and effects of different types of bullying?” and “What are the different patterns in the causes and effects of bullying between genders?”

The process of causal discovery is as follows: (1) defining the key variables; (2) searching for potential causes and effects, (3) collecting or measuring the relevant data; (4) selecting an appropriate causal discovery algorithm and integrating prior knowledge into the causal network; (5) applying the algorithm to the dataset to uncover the causal structure and (6) validating the causal discovery results (Quintana, 2023). Given that defining variables and collecting relevant data depends on expert and domain knowledge, this tutorial focuses on steps (3) to (6).

The required packages for causal discovery are *pcalg* (Kalisch et al., 2012) and *bnlearn* (Scutari, 2010). Additionally, the multiple imputation method from *mice* package (Zhang, 2016) was used to deal with missing data. The *reshape2* package was loaded to reshape data structure (Wickham, 2020) The *ggplot2* (Wickham et al., 2016) and *igraph* (Csardi, 2013) package was loaded to visualize the causal discovery results. The R version is 4.4.1. The code used to analyze the HBSC dataset as well as a simulated example can be found at: https://osf.io/fqjuw/?view_only=8333befd0dec443aa7b07e43ccc468b7.

## Prepare the data

In the HBSC dataset, 36 bullying-related variables were identified from previous studies, covering responses from 93 items (Hertz et al., 2015; Klein et al., 2012; Lereya et al., 2013; Lee et al., 2020; Renshaw et al., 2016; Pulido et al., 2019; Moon et al., 2016; Vieno et al., 2011; Jochman et al., 2017; Jeong & Lee, 2013; Luk et al., 2010; Seeds et al., 2010; Smalley et al., 2017; Stone & Carlisle, 2017; Terzian et al., 2011; Wang et al., 2009a, 2009b, 2010, 2011a, 2011b, 2012; Elgar et al., 2009). These variables were classified into seven categories: demographic, family-related, peer-related, school-related, unhealthy or risky behavior, bullying involvement, and potential bullying outcomes (see Appendix A).

The data was first imported into R for imputation of missing values. We selected random forest imputation given its ability to deal with various data types and distributions (Geurts et al., 2009; Shah et al., 2014). This method’s efficacy has been validated in psychological research contexts (Golino & Gomes, 2016).

After imputation, composite scores for the variables were calculated. For single-item variables, the final score was the item’s value, while for multi-item variables, scores were aggregated by summing items, except for father/mother/friend communication and family structure. Father communication includes two items that assess communication with either a father or stepfather. Mother communication includes two items that measure communication with either a mother or stepmother. Friend communication includes three items assessing communication with a best friend, a same-sex friend, and an opposite-sex friend. For these variables, the highest score among the items was selected, as communication with one person in each category may suffice. Family structure was coded as 1 (both parents present) or 0 (single-parent household) to distinguish between two-parent and single-parent families.

Histograms were then generated to assess the normality of the variables (see Appendix B). Moreover, many studies have indicated that bullying mechanisms may differ by gender (Wang et al., 2009a, 2009b, 2010, 2011a, 2012). Boys and girls can be victims of different types of bullying, and the causal network predicting their bullying victimization can differ substantially. Thus, this study will examine the mechanisms of male and female bullying separately. To conduct this analysis, the dataset was divided by gender.

## Select and implement the algorithm

The selection of algorithms is based on three factors: data distribution, the presence of potential confounders, and the need for incorporation of prior knowledge. For data distribution, the algorithms such as PC, FCI, and MMHC can accommodate both non-normal and normal distributions. While GES often defaulted to a Gaussian BIC score, the algorithm itself is agnostic to distributional form; other data types can be accommodated by specifying or customizing an appropriate BIC. LiNGAM is more suited for non-normal distributed data. Regarding potential confounders, if the dataset contains a limited number of variables or there are known unmeasured confounders, the sufficiency assumption is likely violated. In such cases, algorithms that do not require sufficiency assumptions (e.g., FCI) are preferred. Conversely, when the data is believed to include most of the relevant variables in the causal network, other algorithms can also be suitable. With respect to prior knowledge, many algorithms, such as the PC-Stable and MMHC algorithms in the *pcalg* package, and the PC, FCI, and GES algorithms in the *bnlearn* package, allow users to incorporate prior knowledge to forbid or enforce causal effects from one node to another. This can be achieved using the fixedGaps (to forbid an edge) and fixedEdges (to enforce an edge) arguments in *pcalg*, or blacklists (to forbid an edge) and whitelists (to enforce an edge) arguments in *bnlearn*. The LiNGAM algorithm does not support the use of prior knowledge as input.

We selected LiNGAM as the primary algorithm for our empirical demonstration for two main reasons. First, LiNGAM makes use of the non-Gaussian distribution of observed variables to decide causal direction. Since most variables in the HBSC dataset deviate from normality, LiNGAM is more appropriate than algorithms such as GES or FGES. Second, LiNGAM has the advantage of producing a uniquely identifiable causal DAG. This helps avoid the problem of Markov equivalence, which often limits the ability of conditional-independence-based methods such as the PC algorithm to reveal clear causal directions. These two features make LiNGAM a suitable choice for exploratory analysis of complex psychological data such as the HBSC.

The LiNGAM algorithms use a data matrix as input, with rows representing participants and columns representing variables. The corresponding code for executing these algorithms is provided below.

#Perform LiNGAM algorith m lingam_fit_male < −lingam(as.matrix(data_male)) lingam_fit_female < −lingam(as.matrix(data_female))

## Conduct subgroup analysis

Once the data and parameters are finalized, causal discovery algorithms generate a causal network graph as their output. However, for datasets with many variables, these graphs can become complex and difficult to interpret. Therefore, we recommend extracting the adjacency matrix from the algorithms and using a heatmap to identify causal networks of interest initially.

The adjacency matrix is a matrix representation of a causal graph. Its rows indicate causes and columns indicate effects. Each cell reflects the causal effect of variable X (in the *i*th row) on variable Y (in the* j*th column). For example, a 1 in cell [1, 2] indicates that the first variable has a causal effect on the second variable. The adjacency matrix is directly accessible for all algorithms, except LiNGAM. The LiNGAM algorithm does not directly generate an adjacency matrix. Instead, it produces a matrix containing the coefficients of each causal effect. By recoding non-zero coefficients as indicating the presence of a causal effect and zero coefficients as indicating its absence, an adjacency matrix can be constructed. The following codes show how to get the adjacency matrix from different algorithms:

#Extract coefficient and adjacency matrix from LiNGAM algorithm coef _matrix_lingam < −lingam_fit$B adj_matrix_lingam < −coef _matrix_lingam! = 0

**Fig. 3:**
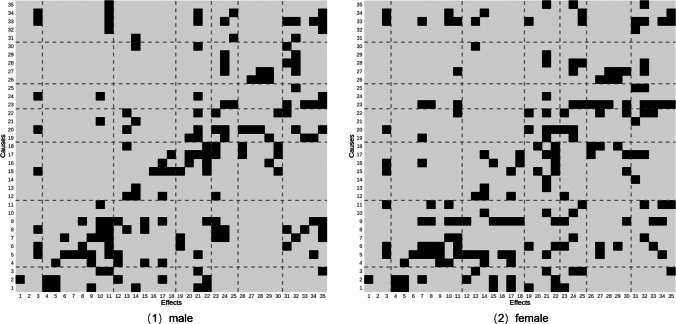
Adjacency matrix from LiNGAM algorithm. *Note.* Black indicates the presence of a causal relationship and grey indicates the absence of a causal relationship. *Dashed lines* divide variables into seven categories: demographic, family-related, peer-related, school-related, unhealthy or risky behavior, bullying involvement, and potential outcomes. The variables names are as follows: 1: Age; 2: Grade; 3: SES; 4: Sibling; 5: Family structure; 6: Father communication; 7: Mother communication; 8: Father monitoring; 9: Mother monitoring; 10: Parent support; 11: Family satisfaction; 12: Computer use; 13: TV use; 14: Junk food; 15: Alcohol use; 16: Smoke use; 17: Fight involvement, 18: Weapons carry; 19: Friend communication; 20: Friends numbers; 21: Isolation; 22: Delinquent friends; 23: Attitude toward school; 24: Classroom environment; 25: Schoolwork pressure; 26: Physical bullying; 27: Verbal bullying; 28: Relational bullying; 29: Cyber bullying; 30: Bullying others; 31: Somatic symptoms; 32: Depressive symptoms; 33: Health condition; 34: Academic performance; 35: Life satisfaction.

**Fig. 4:**
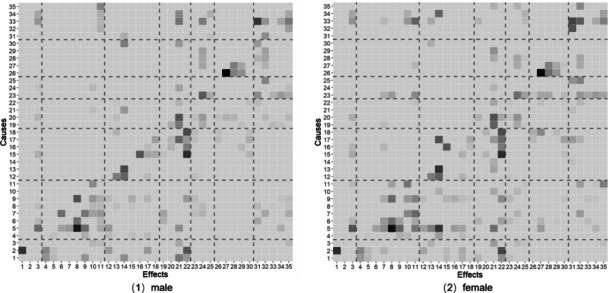
Coefficient matrix from LiNGAM algorithm. *Note.* Darker colors represent stronger effects. *Dashed lines* divide variables into seven categories: demographic, family-related, peer-related, school-related, unhealthy or risky behavior, bullying involvement, and potential outcomes. The variables names are as follows: 1: Age; 2: Grade; 3: SES; 4: Sibling; 5: Family structure; 6: Father communication; 7: Mother communication; 8: Father monitoring; 9: Mother monitoring; 10: Parent support; 11: Family satisfaction; 12: Computer use; 13: TV use; 14: Junk food; 15: Alcohol use; 16: Smoke use; 17: Fight involvement, 18: Weapons carry; 19: Friend communication; 20: Friends numbers; 21: Isolation; 22: Delinquent friends; 23: Attitude toward school; 24: Classroom environment; 25: Schoolwork pressure; 26: Physical bullying; 27: Verbal bullying; 28: Relational bullying; 29: Cyber bullying; 30: Bullying others; 31: Somatic symptoms; 32: Depressive symptoms; 33: Health condition; 34: Academic performance; 35: Life satisfaction.

We can then generate heatmaps using the adjacency matrix (see Fig. 3) and coefficient matrix (see Fig. 4), with the *x*-axis representing effects and the *y*-axis representing causes. In Fig. 3, black cells present the presence of a causal effect, and grey cells present the absence of a causal effect. This heatmap offers a clear visualization of cause-and-effect patterns, facilitating the identification of causes or effects for specific variables. For example, if we are interested in the potential causes of physical bullying (variable 26) among females, we locate 26 on the *x*-axis and identify dark cells within its column. Rows 17, 18, and 23 are dark, corresponding to fight involvement, carrying weapons, and having delinquent friends. This suggests that being a victim of physical bullying might be associated with risky behaviors such as fight involvement, carrying weapons, and delinquent friends, consistent with previous studies on the association between peer environment and bullying victimization (Perren & Hornung, 2005; Walters & Espelage, 2023). In addition to the presence or absence of causal effects, Fig. 4 also illustrates their strength.

The heatmap can also offer a descriptive comparison of bullying patterns across subgroups. For example, to explore gender differences in the direct causes of physical bullying, researchers can compare the adjacency matrices for males and females. This comparison revealed that physical bullying victimization is influenced by fight involvement (variable 17) and weapons carry (variable 18) in both genders. However, females are additionally influenced by delinquent friends (variable 23) while males are additionally influenced by friend numbers (variable 20). This causal-structure difference can serve as a template for future subgroup analyses.

Afterwards, we calculated the Structural Hamming Distance (SHD) and Structural Intervention Distance (SID) as indicators of the difference between the two causal graphs. This was done by hammingDist and structIntervDist functions in the *SID* package (Gruber, 2023; Peters & Bühlmann, 2015). SHD quantifies the number of edge insertions, deletions, or reversals required to transform one graph into another, measuring structural similarity (Acid & de Campos, 2003; Tsamardinos et al., 2006a, 2006b). In contrast, SID assesses the divergence in causal implications by comparing how interventions affect different variables through each graph (Peters & Bühlmann, 2015). To evaluate whether the observed differences in SHD and SID were statistically significant, we employed a permutation test (Good, 2013; Van Borkulo et al., 2023). This approach involves randomly reassigning group labels and recalculating the SHD and SID for each permutation, generating a null distribution of differences under the assumption of no group-level causal differences. The observed SHD and SID values were then compared to this null distribution to obtain empirical p-values. The comparison between the causal graphs of genders revealed an SHD of 143 and an SID of 906. Permutation testing indicated that neither difference was statistically significant, with *p* values of.136 (SHD) and.467 (SID), respectively (see Appendix C for the null distributions). This result indicates no significant difference between subgroups at the level of the overall causal structure. Nonetheless, the previously noted differences require replication to adjudicate whether they represent true structural difference or random error (Hoekstra et al., 2023).

#Calculate the SHD and SID between two causal graph observed_shd < −hammingDist(adj_male, adj_female) observed_sid < −structIntervDist(adj_male, adj_female)$sid

## Construct causal graphs of interest

**Fig. 5:**
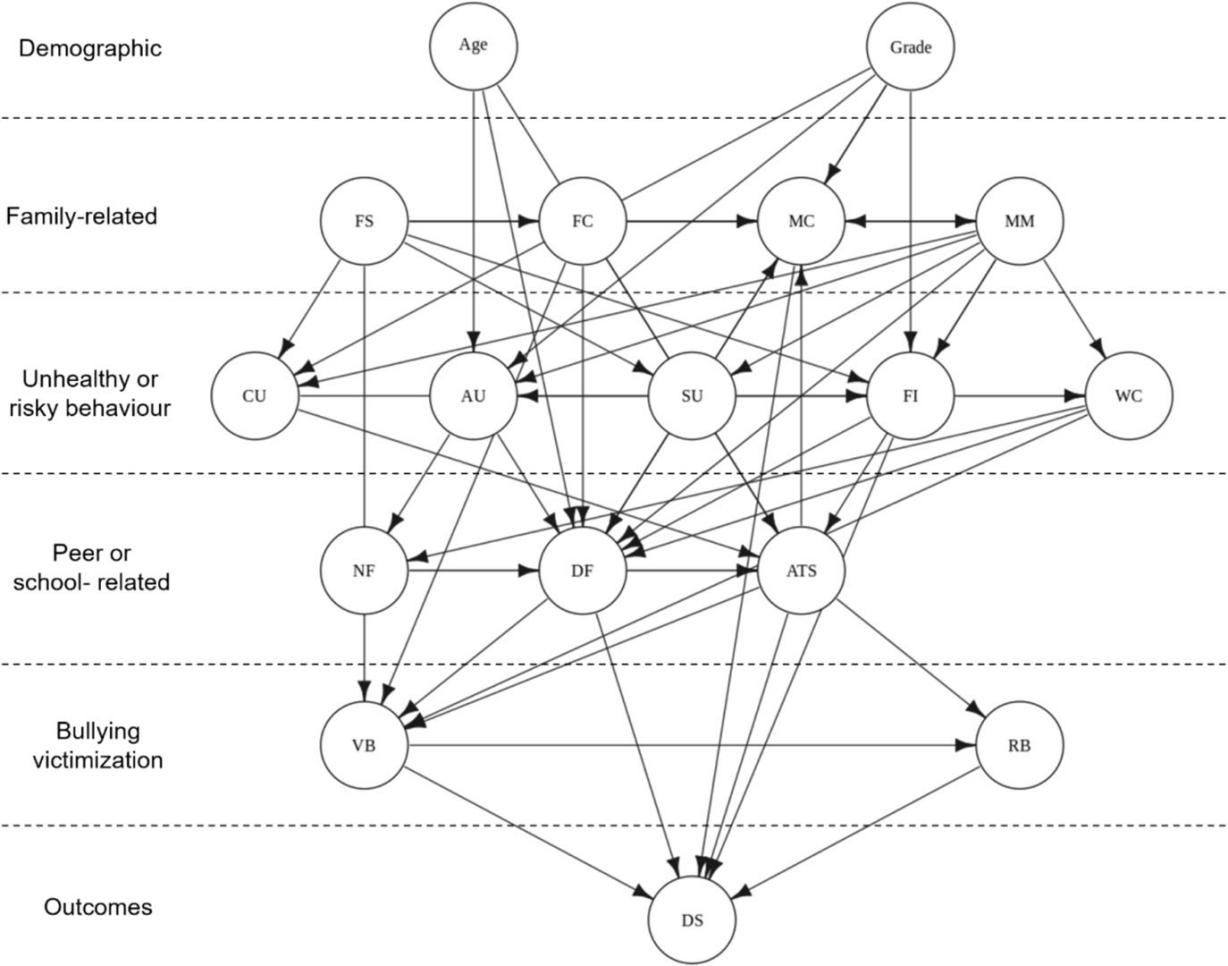
Causal network graph for depressive symptoms in female. *Note.* FS: Family structure; FC: Father communication; MC: Mother communication; MM: Mother monitoring; CU: computer use; AU: Alcohol use; SU: Smoker use; FI: Fight involvement; WC; Weapon carry; NF: Number of friends; DF: Delinquent friends; ATS: Attitude toward school; VB: Verbal bullying; RB: Relational bullying; DS: Depressive symptoms.

**Fig. 6:**
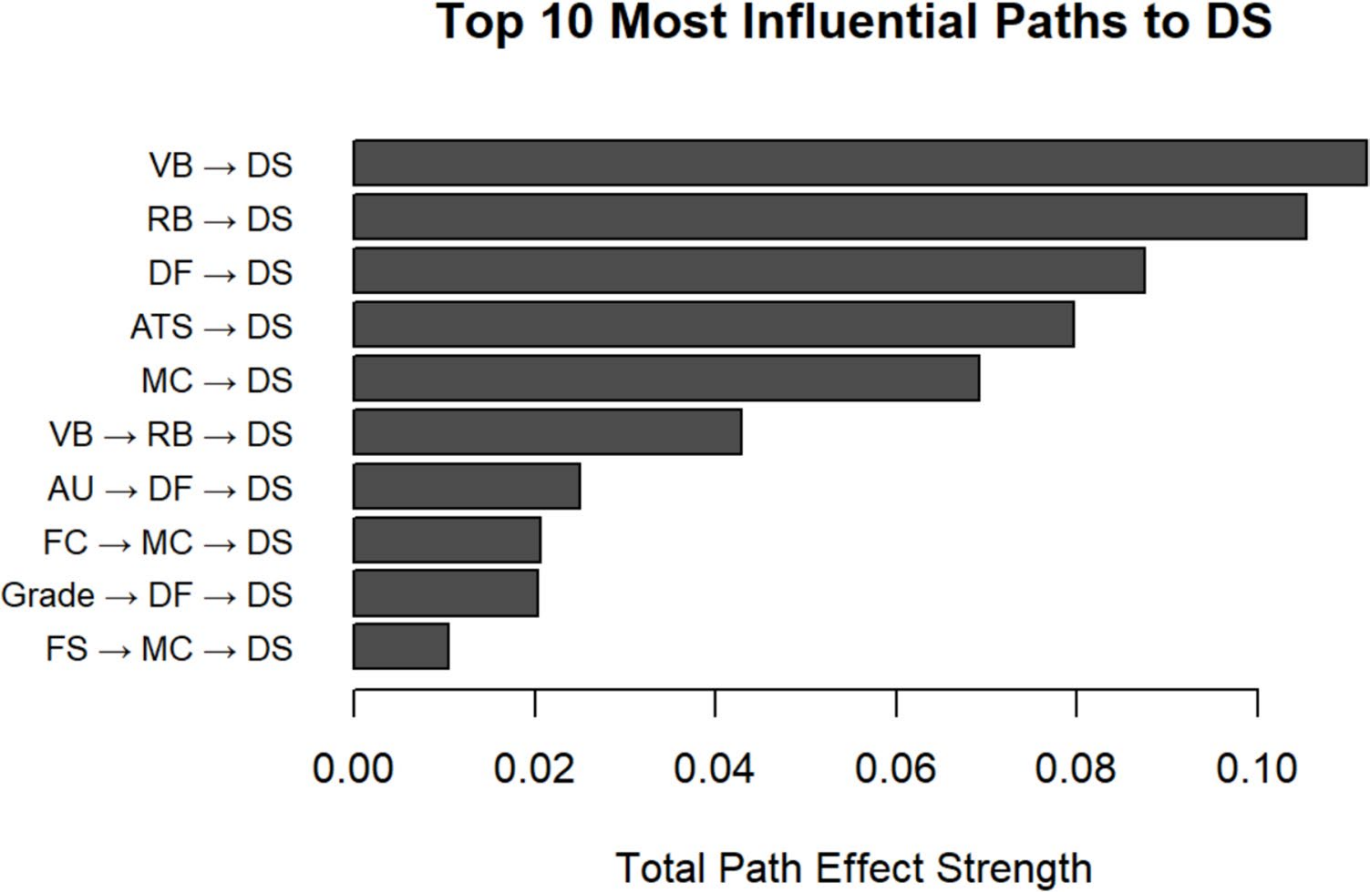
Top 10 most influential pathways to depressive symptoms in female. *Note.* FS: Family structure; FC: Father communication; MC: Mother communication; WC; Weapon carry; DF: Delinquent friends; ATS: Attitude toward school; VB: Verbal bullying; RB: Relational bullying; DS: Depressive symptoms.

The next step is to construct causal graphs of interest. Starting with a particular variable, we can build a preliminary causal network by iteratively identifying its parents and ancestors. The causal graph can be generated using the *igraph* package (Csardi, 2013). Packages such as *NetworkD3* (Allaire et al., 2017) and *visnetwork* (Almende et al., 2019) also enable the creation of interactive causal graphs. For example, to investigate the bullying-related causal network of depressive symptoms (variable 32) in females, we traced its parent nodes within the bullying category—verbal bullying (variable 27) and relational bullying (variable 28)—and mapped their respective ancestors. This analysis revealed a multi-layered causal graph involving demographic, family, peer, and risky behavioral factors (see Fig. 5). Driven from this graph, we can also quantify the strength of each causal pathway by multiplying the coefficients along the path and identifying the top 10 most influential pathways leading to depressive symptoms, as shown in Fig. 6.

It is worth noting that the validity of the inferred causal network depends on the key assumptions, including linearity, sufficiency, faithfulness, and acyclicity. As such, the resulting graph should be regarded as a hypothesis-generating template rather than a definitive causal model. Moreover, in constraint-based methods such as PC and FCI, the causal graphs should not be automatically interpreted as representing a mixture of direct and indirect effects, since the edges are inferred based on the d-separation criterion and only reflect conditional dependence relationships.

To assess the importance of variables within the network, the *igraph* package also provides tools to compute centrality measures (Epskamp et al., 2018). These centrality measures include, but are not limited to: (1) betweenness, which quantifies how often a node lies on the shortest path between other nodes; (2) closeness, which reflects average length of pathway a node is connected to all other nodes in the network; and (3) eigenvector, which captures how frequently a node is connected to other influential nodes (nodes with more edges).

**Fig. 7:**
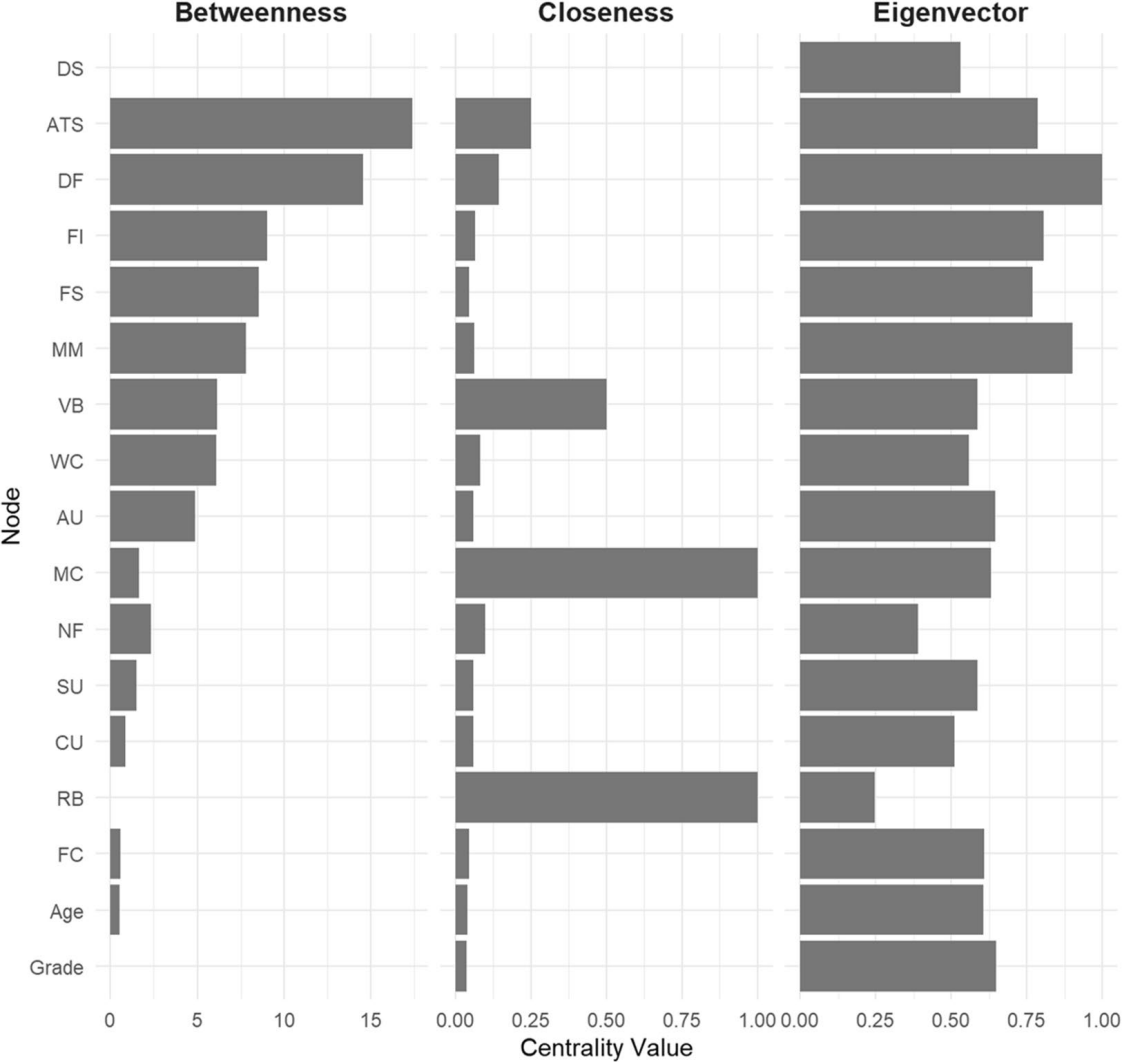
Centrality measures of each node within the predefined networks related to depressive symptoms. *Note.* FS: Family structure; FC: Father communication; MC: Mother communication; MM: Mother monitoring; CU: computer use; AU: Alcohol use; SU: Smoker use; FI: Fight involvement; WC; Weapon carry; NF: Number of friends; DF: Delinquent friends; ATS: Attitude toward school; VB: Verbal bullying; RB: Relational bullying; DS: Depressive symptoms. *Nodes without bars* indicate that the centrality index could not be calculated for those nodes, either because they only serve as causes or do not lie on any relevant pathways.

Figure 7 presents the results of these centrality measures within our predefined networks related to depressive symptoms. Attitudes toward school and associations with delinquent friends were identified as nodes with the highest betweenness, indicating their potential role as key mediators within the network. In contrast, mother communication, verbal bullying, and relational bullying were identified as the nodes closest to others, suggesting that they are the most central variables associated with teenagers’ depressive symptoms.

#Betweenness centrality betweenness(g) #Closeness centrality closeness(g) #Eigenvector centrality eigen_centrality(g)$vector

## Validate the results from causal discovery algorithms

While causal discovery algorithms can effectively search for possible causal networks, judgments regarding the presence or absence of causal relationships can be influenced by noise during data collection (Colombo & Maathuis, 2014; Hoekstra et al., 2023). Thus, several approaches can be employed to increase the stability of the results and enhance confidence in findings.

The first approach is to bootstrap the algorithm and test the stability of edges across different samples (Briganti et al., 2023; Meinshausen & Bühlmann, 2010). The rationale is that if a causal relationship is replicated in most subsamples, the causal pathway can be considered robust to potential noise. This approach can be implemented in R by repeatedly sampling from the dataset, running the algorithm on each subsample, and calculating the frequency of identified causal pathways. Additionally, packages such as *bnlearn* also provide their own bootstrap function. Briganti et al. (2023) recommended that researchers retain only those edges that appear in more than 85% of samples and whose directions are consistent in over 50% of samples.

The second approach is to validate causal discovery results across multiple algorithms. By applying different causal discovery methods to the same dataset and identifying the structure they have in common, researchers can obtain a more robust estimate of the underlying causal graph. This ensemble approach is less sensitive to sampling variability and noise, and helps mitigate the risk of drawing misleading conclusions based on the assumptions of a single method (Volkova et al., 2023). The rationale is that a true causal structure should be compatible with the assumptions of multiple algorithms; therefore, if an edge reflects an underlying causal relationship, it is more likely to be recovered consistently across different methods. Several studies have shown that extracting consensus graphs—by including only edges that appear in the majority of methods—can improve the reliability and precision of the inferred structure (Ma et al., 2023; Sinha et al., 2021; Wen et al., 2022; Zhang et al., 2024). A common criterion in constructing consensus graphs is to retain edges that appear in more than 50% of the individual graphs—a procedure known as the majority voting rule (Wen et al., 2022; Zhang et al., 2024). Consensus graphs can also be used to assess the relative performance of different causal discovery algorithms through a method known as intersectional validation (Viinikka et al., 2018). In a simulation study, Viinikka and colleagues showed that consensus graphs can closely approximate the ground-truth structure, making them a useful benchmark when the true model is unknown. Following this approach, Appendix D provides a comparison of LiNGAM, GES, and MMHC as applied to our tutorial dataset.

The third approach is to test specific features of the causal structures identified by discovery algorithms. One option is to examine whether the conditional independence relationships implied by the graph hold in the observed data, using the d-separation criterion. This is particularly relevant when the graph is generated by methods that do not rely on conditional independence as part of their search procedure, such as score-based or asymmetry-based algorithms. Software packages such as *bnlearn* provide a range of conditional independence tests that can be used for this purpose. Prior studies have shown that evaluating d-separation relations can help identify spurious edges and improve the overall credibility of the inferred structure (Eulig et al., 2025; Ramsey et al., 2024). This type of structural check offers a way to assess whether the graph is consistent with the empirical data.

The fourth approach is to intervene on the target variable and use interventional data to differentiate and validate candidate causal graphs (He & Geng, 2008). Causal discovery from purely observational data is fundamentally constrained by Markov equivalence, where multiple causal graphs may share the same set of conditional independence and cannot be distinguished without further information. Active interventions offer a solution by breaking the conditional dependencies. By setting the value of a variable externally (hard intervention) or influencing the distribution of a certain variable (soft intervention), one can observe subsequent changes in the child variables and isolate causal effects from conditional dependencies (He et al., 2008). For example, given three variables (A, B, and C), intervening on a single variable—such as A—and observing the resulting changes in the others can be sufficient to distinguish between chain, fork, and collider structures in a causal graph.

However, in real-world scenarios, interventions are often costly, risky, noisy, or imprecise. To address the potential real-world constraint, several methods have been proposed to identify a finite set of interventions that maximize information gain and reduce posterior model uncertainty under resource constraints (e.g., Agrawal et al., 2019; Mukherjee et al., 2024; Tigas et al., 2022; Zemla & Miller, 2023). Meanwhile, a growing body of work has also begun to address cases where the targets of intervention are imprecise, noisy, or entirely unknown (e.g., Choo et al., 2024; Ke et al., 2019; Mascaro & Castelletti, 2023). For example, Ke et al. (2019) introduced a neural causal model that automatically identifies which variables have been intervened upon and uses this inferred intervention information to distinguish candidate causal structures.

Furthermore, when direct intervention is too risky or infeasible—such as in real-world driving environments—counterfactual simulation has been proposed as an alternative approach to support causal discovery (Howard & Kunze, 2023). One such strategy involves removing or controlling a node within a hypothesized causal chain and observing the resulting changes in downstream events. If the outcome disappears or changes significantly in the absence of the node, this provides evidence that the removed node was a necessary cause of the downstream events.

The fifth approach is to corroborate causal discovery results with prior knowledge or other sources of evidence (Briganti et al., 2023; Gradu et al., 2024; Yarkoni, 2020). Causal discovery algorithms do not distinguish between endogenous and exogenous variables, which can lead to implausible causal relationships that contradict prior knowledge (Nogueira et al., 2022). Those implausible causal relationships can be excluded by incorporating prior knowledge directly into the algorithm or applying it post hoc to refine the results. Meanwhile, psychological theory as well as experts’ knowledge can be used to support or explain the psychological mechanism behind the identified causal relationship (Yarkoni, 2020). Qualitative research, such as interviews, can also help make sense of the observed causal relationship through participants’ subjective experiences (Pekrun et al., 2002).

These approaches can be used in combination to strengthen the validity of causal discovery results and increase confidence in the inferred structures.

## Discussion

This paper introduces the concepts and algorithms of causal discovery and provides a tutorial on analyzing psychological data using these methods. The tutorial demonstrates the capability of causal discovery algorithms in uncovering causal networks from observational data, as well as their ability to identify the different causal networks between subgroups.

As mentioned at the outset, causal discovery methods represent a promising approach to discovering causal relationships from observational or interventional data and can complement or accelerate theoretical development (Meehl, 1978). Psychologists have traditionally refrained from explicitly stating causal relationships when working with observational data (Grosz et al., 2020). However, their assumptions about causality are often implicit in the way they describe mechanisms and select predictors and outcome variables. Without clear causal criteria, studies based on correlational and regression analyses could provide supporting evidence for both directions, which would confuse practical recommendations and implications. For example, the causal relationship between risky behaviors and bullying victimization has been a subject of debate (e.g., Holt et al., 2013; Innamorati et al., 2018; Litwiller & Brausch, 2013; Poon, 2016; Shah et al., 2022; Terzian et al., 2011), with some arguing that individuals with a high tendency for risk-seeking are more likely to place themselves in situations where they may become victims of bullying, and others arguing that teenagers engage in risky behaviors as a coping mechanism to deal with the stress of being bullied. The analysis of the HBSC dataset in the current tutorial provides evidence that risky behavior is more likely to be the cause, rather than the effect, of bullying victimization. It also revealed that exposure to delinquent friends is a mediator in this causal relationship.

Moreover, causal discovery methods can be used to guide precise interventions targeting psychological phenomena (Bronstein et al., 2024). Precise intervention is a targeted, specific approach designed to maximize the efficiency of actions (e.g., policies or treatments) while minimizing resource use (Deisenhofer et al., 2024; Mannion, 2022; Deisenhofer et al., 2024; Mauch et al., 2022). Developing such interventions often requires a comprehensive understanding of the phenomena, including their underlying mechanisms, the category of subtypes, and the interplay of individual, social, and environmental factors (Georas et al., 2022; Mauch et al., 2022). However, achieving this level of insight can be challenging with traditional psychological theories or experiments alone (Deisenhofer et al., 2024). As demonstrated in this tutorial, causal discovery methods can aid the precise intervention by outlining the causal networks and identifying highly influential nodes within causal networks. Meanwhile, these methods enable the exploration of causal networks across different subgroups, which can be leveraged to design targeted interventions for specific populations (Anderson et al., 2023; Fung et al., 2023).

While causal discovery methods present exciting opportunities for advancing psychological research, they also come with challenges, underscoring the need for further adaptation to address the unique requirements of the field. For one, the validity of causal discovery results relies on the assumptions regarding acyclicity, faithfulness, and sufficiency, among which one of the most significant challenges is eliminating the potential presence of confounders. Confounders pose a challenge not only for causal inference using traditional statistical approaches such as regression and structural equation modeling (SEM; Stuart et al., 2021), but also for causal discovery methods (Chen et al., 2021). As such, causal discovery methods are not a one-size-fits-all solution for establishing causality (Binder et al., 1997). Instead, researchers are encouraged to integrate causal discovery findings with established causal evidence and randomized experiments to enhance the credibility of these findings (Briganti et al., 2023). Additionally, to evaluate the influence of potential confounders on their analysis, researchers usually use sensitivity tests to simulate the effect of confounders and determine how strong the effect would need to be to change the statistical results meaningfully (Imai et al., 2010; Robins et al., 2000). Yet, as far as we know, no sensitivity test exists specifically for causal discovery methods. Future research could address this gap.

Second, as outlined earlier in the Causal Discovery Algorithms section, the statistical power of causal discovery is influenced by multiple factors. Causal discovery methods can accommodate many different data types, but the required sample size varies a lot among different data types. Briganti et al. (2023) have suggested using power and sample size calculations for linear and logistic regressions as a proxy (e.g., Bonett & Wright, 2000). However, the required sample size also depends on the number of nodes and edges in the causal network. Larger networks with more nodes and edges require proportionally larger samples to maintain the statistical power and accuracy of causal discovery methods (Tashiro et al., 2014; Xu et al., 2019). Kummerfeld and colleagues (2024) have developed a power analysis tool for PC and GES algorithms (https://kummerfeldlab.shinyapps.io/PowerSim2023-1/) and researchers can use it as a guideline for estimating the required sample size for their analyses, but there remains a lack of a comprehensive sample size estimation tool for causal discovery methods more generally.

Third, the challenges posed by heterogeneous data and data fusion in causal discovery studies should not be overlooked. Unlike ideal settings that assume data are independently and identically distributed, and originate from a single observational or interventional source, real-world data are often fragmented and non-stationary. On the one hand, heterogeneous data are often collected from multiple environments that vary by time, location, or population, each potentially introducing different levels of measurement error and noise (Günther et al., 2023; Jalaldoust et al., 2025). The underlying causal mechanisms themselves may also differ across these subgroups (Huang et al., 2020; Perry et al., 2022; Zhang et al., 2017). In such cases, conditional (in)dependence relations observed in a single environment may fail to generalize across contexts, rendering them unreliable indicators of the true causal structure. On the other hand, real-world datasets are often hybrid in nature, containing a mix of continuous and discrete variables, as well as varying degrees of missing data (Sokolova et al., 2017). Even datasets nominally addressing the same domain may measure overlapping but non-identical sets of variables (Li et al., 2023). These issues give rise to the data fusion problem: how to recover a coherent and complete causal graph from partially observed and heterogeneous causal relationships.

Regarding the first challenge, the CD-NOD framework has been proposed to address it. It detects shifts in variable distributions across environments and uses the shifts, along with induced changes in other variables, as signals for identifying causal directions (Huang et al., 2020; Perry et al., 2022; Zhang et al., 2017). Regarding the second challenge, Sokolova et al. (2017) proposed the use of copula-based conditional independence tests to overcome the challenge posed by mixed data types. They also recommended addressing missing values by combining imputation techniques with multiple independence tests and applying a majority voting strategy to select stable edges. Meanwhile, Li et al. (2023) explored the use of federated learning to aggregate locally inferred causal graphs from disparate datasets to construct a unified global causal graph. Together, these approaches can effectively address the challenges of causal discovery under real-world data constraints.

Finally, future studies could investigate how to better incorporate prior expert knowledge into the causal criteria of these algorithms. For example, causal discovery methods can be combined with machine learning algorithms to further enhance its ability to uncover causal relationships from psychological data. This allows the algorithms to actively learn patterns of causal relationships from empirical data and prior knowledge (Lagemann et al., 2023) and can refine the estimation of causal strengths (Brand et al., 2023; Feuerriegel et al., 2024; Zhao & Liu, 2023). Meanwhile, the application of machine learning algorithms in causal strength estimation allows prediction of individual treatment outcomes based on patients’ profiles (Feuerriegel et al., 2024). This method holds promise for the development of precise interventions using causal discovery methods.

## Conclusion

In conclusion, causal discovery methods are powerful tools for uncovering causal relationships from observational and interventional data. These methods can significantly accelerate the discovery of causal relationships in psychological research, thereby advancing our understanding of psychological phenomena and the human mind. While challenges remain in applying causal discovery to psychology, the opportunities far outweigh the obstacles. We encourage researchers to contribute to the validation of these methods and apply them broadly across psychology.

## Figures and Tables

**Fig. B1:**
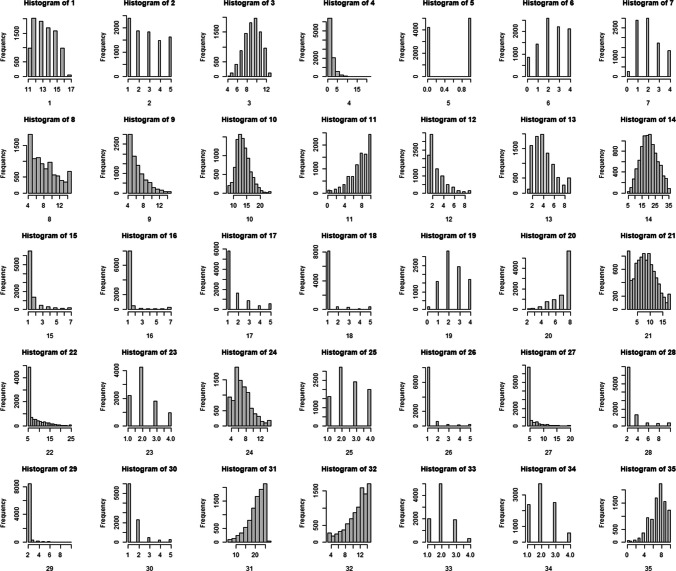
Histogram of data. *Note.* 1: Age; 2: Grade; 3: SES; 4: Sibling; 5: Family structure; 6: Father communication; 7: Mother communication; 8: Father monitoring; 9: Mother monitoring; 10: Parent support; 11: Family satisfaction; 12: Computer use; 13: TV use; 14: Junk food; 15: Alcohol use; 16: Smoke use; 17: Fight involvement, 18: Weapons carry; 19: Friend communication; 20: Friends numbers; 21: Isolation; 22: Delinquent friends; 23: Attitude toward school; 24: Classroom environment; 25: Schoolwork pressure; 26: Physical bullying; 27: Verbal bullying; 28: Relational bullying; 29: Cyber bullying; 30: Bullying others; 31: Somatic symptoms; 32: Depressive symptoms; 33: Health condition; 34: Academic performance; 35: Life satisfaction

**Fig. C1:**
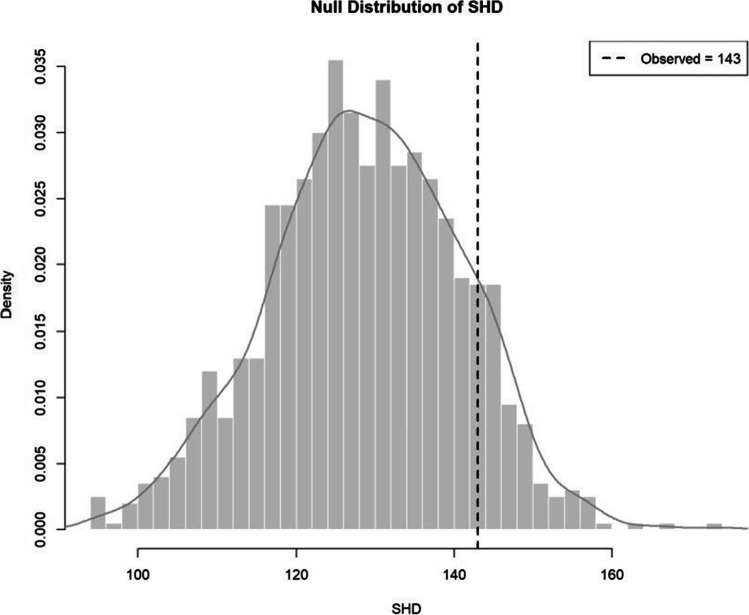
Permutation test on SHD

**Fig. C2:**
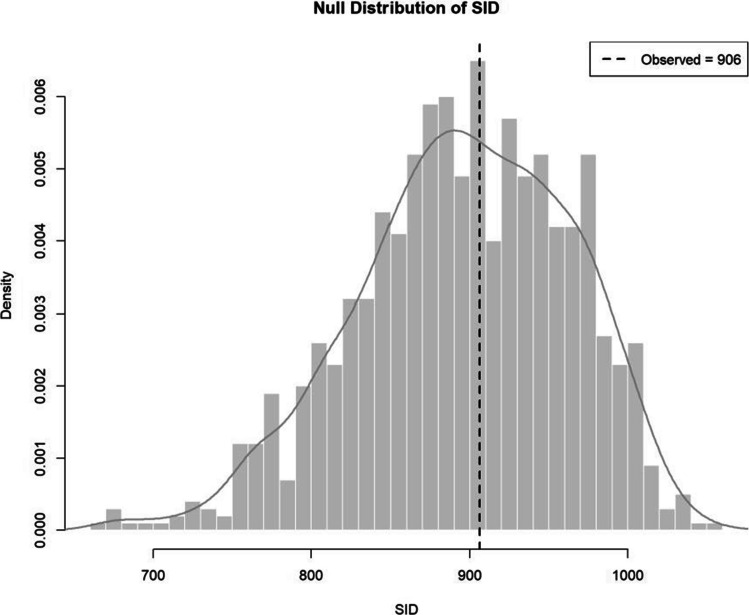
Permutation test on SID

## Data Availability

The datasets used in this tutorial are available from https://www.icpsr.umich.edu/web/NAHDAP/studies/28241

## Appendix A

**Table A1:** Variables of interest

Category	Name	Corresponding column in dataset
Demographic	Gender	Q1
	Age	AGE
	Grade	Q4
	SES	Q12-15
Family-related	Family structure	Q16_1, Q16_2
	Sibling numbers	Q16_9, Q16_10
	Father communication	Q47A, Q47B
	Mother communication	Q47C, Q47D
	Mother monitoring	Q48A-E
	Father monitoring	Q49A-E
	Parent support	Q50A-H
	Family satisfaction	Q51
Peer-related	Talk with friends	Q47G
	Friend numbers	Q52_1, Q52_2
	Isolation from friends	Q54-56
	Delinquent friends	Q74A-E
School-related	Attitude toward school	Q59
	Classroom support	Q60
	Schoolwork pressure	Q61
Unhealthy or risky behavior	Computer use	Q9
	TV use	Q18
	Junk food	Q25C, Q25D, Q25M, Q25N, Q26
	Fight	Q66
	Weapon	Q67
	Smoke	Q72A
	Alcohol	Q72B
Bullying involvement	Physical bullying	Q63C
	Verbal bullying	Q63A, Q63E, Q63F, Q63G
	Relational bullying	Q63B, Q63D
	Cyber bullying	Q63H, Q63I
	Bullying others	Q64
Bullying outcomes	Life satisfaction	Q7
	Health	Q44
	Academic performance	Q58
	Somatic symptoms	Q39A, Q39B, Q39C, Q40C, Q45D
	Depressive symptoms	Q39D, Q45A, Q45C

## Appendix D

Using the intersectional-validation approach, we applied LiNGAM, GES, and MMHC to the same dataset, each bootstrapped 100 times, and retained only edges that appeared in more than 50 iterations. We then constructed a consensus graph by identifying the common structures across all results. The causal graphs produced by the three algorithms were compared to the consensus graph using Structural Hamming Distance (SHD) and Structural Intervention Distance (SID). As shown in Table 5, across both male and female subgroups, GES consistently exhibited the greatest divergence from the consensus graph. This pattern is consistent with our earlier discussion that the original GES algorithm assumes Gaussian distributions for continuous variables, which may limit its suitability for psychological data that deviate from normality.

**Table D1:** Divergence from the consensus graph among LiNGAM, GES, and MMHC algorithms

	Male				Female			
		LiNGAM	GES	MMHC		LiNGAM	GES	MMHC
Distance	SHD	104	138	104	SHD	91	126	79
	SID	0	0	0	SID	0	16	0

Larger values represent greater divergence.

## Code availability

The code used for analysis during the current study is available from https://osf.io/fqjuw/?view_only=8333befd0dec443aa7b07e43ccc468b7.
